# Perceived stress from social isolation or loneliness among clinical and non-clinical healthcare workers during COVID-19

**DOI:** 10.1186/s12889-024-18363-7

**Published:** 2024-04-11

**Authors:** Katherine A. Meese, Laurence M. Boitet, Katherine L. Sweeney, David A. Rogers

**Affiliations:** 1 Department of Health Services Administration, University of Alabama at Birmingham (UAB), Birmingham, United States; 2 UAB Medicine Office of Wellness, UAB, Birmingham, United States; 3 Department of Medical Education, UAB, Birmingham, United States; 4 Department of Sociology, UAB, Birmingham, United States; 5 Department of Surgery, UAB, Birmingham, United States

**Keywords:** Healthcare, Loneliness, Social isolation, Pandemic, Interprofessional

## Abstract

**Background:**

Workplace social isolation and loneliness have been found to result in a decline in job satisfaction and an increase in burnout among working individuals. The COVID-19 pandemic exacerbated feelings of loneliness and social isolation among healthcare workers. The majority of research on healthcare worker experiences is conducted in siloes which does not reflect the shared experiences of interprofessional teams. The purpose of this study is to understand stress from social isolation or loneliness across the entire clinical and non-clinical healthcare team over the course of the pandemic.

**Methods:**

Data was acquired using a cross-sectional survey distributed to healthcare workers once a year at a large academic medical center in the Southeastern United States during the COVID-19 pandemic (2020–2022). Information pertaining to job role, work location, and demographic factors was collected. Participants were also asked to assess individual well-being and resilience, in addition to reporting stress derived from various sources including job demands and social isolation or loneliness. Descriptive statistics and bivariate analyses were conducted to assess the association between stress from social isolation or loneliness and individual characteristics.

**Results:**

Stress from social isolation or loneliness was found to decrease over the survey period across all measured variables. Trainees and physician-scientists were found to report the highest rates of this stressor compared to other job roles, while Hospital-Based ICU and Non-ICU work locations reported the highest rates of loneliness and social isolation stress. Younger workers and individuals from marginalized gender and racial groups were at greater risk for stress from social isolation or loneliness.

**Conclusions:**

Given the importance of social connections for well-being and job performance, organizations have a responsibility to create conditions and mechanisms to foster social connections. This includes establishing and reinforcing norms of behavior, and developing connection mechanisms, particularly for groups at high risk of loneliness and social isolation.

## Background

Loneliness is defined as the feeling or idea that an individual’s social connections are going unfulfilled, and their needs for belonging are not being sufficiently met [1, 2]. A similar concept, social isolation, occurs when an individual has few or infrequent social interactions and contacts, and can result in the individual experiencing loneliness [3]. The major risk factors for social isolation and loneliness are predisposed physical health complications, psychological and neurological health factors, and socioenvironmental factors [3]. Some of the physical and mental health conditions linked to social isolation and loneliness include, but are not limited to, hypertension, cardiovascular disease, stroke, depression, and suicide [4]. Individuals who are lonely are 26% more likely to die than those who are not lonely [5].

Social isolation and loneliness are best studied through the stress theory’s “buffering model.” The “buffering model” claims that having social support from friends, family, or colleagues, helps protect the physical and mental health of the individual [6]. A lack of social support increases the individual’s amount of social loneliness experienced, which thus increases adverse physical and mental health symptoms. Having the loss of a relationship or lack of close relationship increases the individual’s amount of emotional loneliness experienced, which thus increases adverse physical and mental health symptoms [6]. Workplace loneliness is defined as a mix between emotional loneliness, the lack of a close, high quality connection with another individual, and social loneliness, the lack of having social or casual relationships to share personal interests with others [7]. Workplace social isolation and loneliness have been found to result in a decline in job satisfaction and an increase in burnout among working individuals [8].

In addition to social isolation and loneliness, many healthcare workers also experienced an increase in job demands during the pandemic as heavy patient loads occurred as coworkers were out sick, people left for traveling positions, and others retired early [9]. Job-Demands Resources theory suggests that social resources are an important subset of work-related resources that help offset the effect of job demands. The absence of social connection during the peri-pandemic period may have also left healthcare workers less equipped to address the increase in job demands that occurred during that time [10]. Further, with increased job demands, healthcare workers may have had less time available to seek social support at work.

The majority of research on social isolation or loneliness among healthcare workers has been from 2020, when the COVID-19 global pandemic began, and on. COVID-19 is a major contributing factor of loneliness and social isolation among healthcare workers [11, 12]. When the pandemic began, healthcare workers experienced extreme stress and anxiety about contracting the COVID-19 virus and passing it to their families and friends [13]. In 2020, approximately 76% of healthcare workers reported worrying that they would expose their children to COVID-19, while 47% of healthcare workers reported feeling worried about exposing their family members in general [13]. A 2021 study looked at loneliness among nurses and found that loneliness is significantly correlated to burnout and is a significant predictor of professional quality of life [8]. Healthcare workers experienced a rapid decline in mental well-being during the first year of the pandemic. While healthcare workers mental well-being has increased since many of the CDC guidelines and rules surrounding COVID-19 have been lifted, their well-being is still being reported at much lower levels than their well-being before the pandemic began [14]. Poor well-being has detrimental impacts on individual’s work ethic, professionalism, quality of care, efficiency, and quality of life [15].

Another significant cause of loneliness among healthcare workers, specifically physicians, is the transition into telehealth and virtual care [16]. Telehealth visits have become increasingly more common since the start of the COVID-19 pandemic in 2020, and led to the separation between nurses and patients, and physicians and patients [16]. Healthcare workers were also separated from each other as well, resulting in even more isolation at work. During the peak of the pandemic, four in ten healthcare workers were reporting feeling lonely and isolated [5]. Physicians are reportedly the loneliest out of all healthcare workers. In fact, approximately 45% of family medicine physicians in a national sample report experiencing loneliness [4].

As with much healthcare research, experiences are often studied in professional siloes. This leads to reports of nurse experiences, or physician experiences, but creates a noticeable gap in understanding the shared experiences that occur in the interprofessional teams that characterize our modern healthcare workplace. The purpose of this study is to examine trends in stress caused from loneliness and social isolation over the course of the pandemic, and to identify which healthcare workers are at greatest risk across the entire healthcare team, including clinical support services and administrative and non-clinical workers. Rather than examining the degree of reported social isolation or loneliness, we seek to understand the perceptions of stress caused from these combined phenomena. Further, we seek to understand the association between perceived stress from social isolation and loneliness and well-being, resilience, and job demands.

## Methods

### Data collection and study participants

Employees of a large academic medical center in the southeastern United States completed cross-sectional, anonymous surveys during the month of June 2020, 2021, and 2022. Surveys were distributed by email and reminders were sent weekly during the collection period. Participants were informed of the voluntary nature of the survey and were asked to provide informed consent. If consent was not granted, participants were automatically exited from the survey platform. This study was approved by the surveying institution’s Institutional Review Board.

Participants were asked questions that collected information about individual and organizational level factors, such as burnout, resilience, and well-being, in addition to information related to work and nonwork-related stressors. A total of 10,599 participants completed the annual surveys. Using list-wise deletion, 4,289 cases were deleted due to missing information leaving a final analytic sample of 6,310.

### Dependent variables

Participants were asked to identify major work, clinical, and nonwork-related stressors from a previously generated list [17]. From this list of stressors, social isolation or loneliness was chosen as the outcome variable for the purposes of this study. Participants who indicated social isolation or loneliness as a major nonwork-related stressor were assigned a value of 1, while those who did not were given a value of 0.

#### Independent variables

Participants were asked to provide information related to their job role, work location, gender identity, race, age, and marital status. For the purposes of this study, the sample was limited to those in Administrative and Non-clinical staff, Advanced Practice Provider (APP), Clinical Support Staff, Nurse, Physician or Clinician, Physician Scientist, Trainee (Resident/Fellow), and Other (Basic science and clinical laboratory personnel, social worker, non-clinician faculty, etc.) roles. Work location was represented using Administration/Office, Ambulatory/Outpatient, Hospital-Based Intensive Care Units (ICU), Hospital-Based Non-ICU, Operating Room (OR)/Surgical/Procedural, and Other. To assess gender, participants were asked to select whether they identified as male, female, or non-binary or self-describe. Race was assessed using White, Black or African American, Hispanic or LatinX, Asian or Pacific Islander, or Other/Self-Identify categories. Due to low numbers in Native American or Alaskan Native, Middle Eastern, and Multiracial categories, these identities were combined with the Other category. Participant age was represented in 18–24, 25–34, 35–44, 45–54, 55–64, and greater than 65 categories. Marital status was broken down into Married, partnership, or cohabitating and non-married, partnership, or cohabitating.

Individual resilience was measured using the 2-item Connor-Davidson Resilience Scale (CD-RISC) [18] with which scores that range from 0 to 8 were generated. Overall well-being was evaluated using the 9-item Well-Being Index (WBI) measure [19] and scores were generated that ranged from − 2 to + 9, with scores greater than or equal to 2 indicating distress. A distress variable was generated using WBI scores wherein scores of 2 or greater were assigned a 1, while scores that ranged from − 2 to 1 were assigned a 0. To control for outcomes related to job demands, participants who indicated increased responsibilities or job demands as a major stressor were assigned a value of 1, while those who did not received a value of 0. While other measures were collected during the survey period, the relationship between stress from social isolation and loneliness, demographics and mental health outcomes and job demands were the focus of this present study.

### Data analysis

Categorical variables were represented by frequency and percentages, while numerical data were represented by mean and standard deviation. Chi-squared analyses were used to test the association between the outcome (social isolation or loneliness as a major stressor) and categorical predictors. Bivariate logistic regression was used to test the association between the outcome variable and all numeric variables. Analysis of variances (ANOVA) was used to test the association between continuous outcomes and categorical variables.

## Results

### Description of sample

Response rates were calculated by the number of survey respondents divided by the number of opened emails containing the survey link. This resulted in response rates of 18% in 2020, 46% in 2021, and 27.3 in 2022. Table 1 describes the study sample and includes the frequency and percentage of each demographic category. Participants with job roles designated as “Other” made up the majority of the sample (30.0%), followed by Nurses (18%) and Administrative and Non-clinical staff (17.4%). The dominant work location of the study sample was Administrative Office (28.5%), other work locations (24.7%), and Ambulatory or Outpatient locations (20.7%). Nearly three-quarters of the sample (74.4%) identified as female and white (73.3%). The majority of the sample was 25–34 (27.7%) or 35–44 (24.6%) years old.

**Table 1:** Sample characteristics (*n* = 6,310)

	Freq(%)
Survey Year	
2020	716(11.4)
2021	1,917(30.4)
2022	3,677(58.3)
Job Role	
Administrative and Non-Clinical Staff	1,098(17.4)
APP	421(6.67)
Clinical Support Staff	757(12.0)
Nurse	1,137(18.0)
Physician and Clinical Faculty	699(11.1)
Physician Scientist Faculty	134(2.12)
Trainee (Resident/Fellow)	171(2.71)
Other	1,893(30.0)
Work Location	
Administrative Office	1,799(28.5)
Ambulatory/Outpatient	1,304(20.7)
Hospital-Based ICU	438(6.94)
Hospital-Based Non-ICU	891(14.1)
Operating Room/Surgical/Procedural	322(5.10)
Other	1.556(24.7)
Gender	
Male	1,582(25.2)
Female	4.693(74.4)
Non-binary or Self-describe	25(0.40)
Race	
White	4,628(73.3)
Black or African American	986(15.6)
Hispanic/LatinX	146(2.31)
Asian/Pacific Islander	345(5.47)
Other/Self-Identify	205(3.25)
Age	
18–24	325(5.15)
25–34	1,749(27.7)
35–44	1.549(24.6)
45–54	1.368(21.7)
55–65	1.064(16.9)
> 65	255(4.04)

Note: APP, Advanced Practice Provider; ICU, Intensive Care Unit

#### Social isolation or loneliness as a major stressor by demographic identifiers

**Fig. 1:**
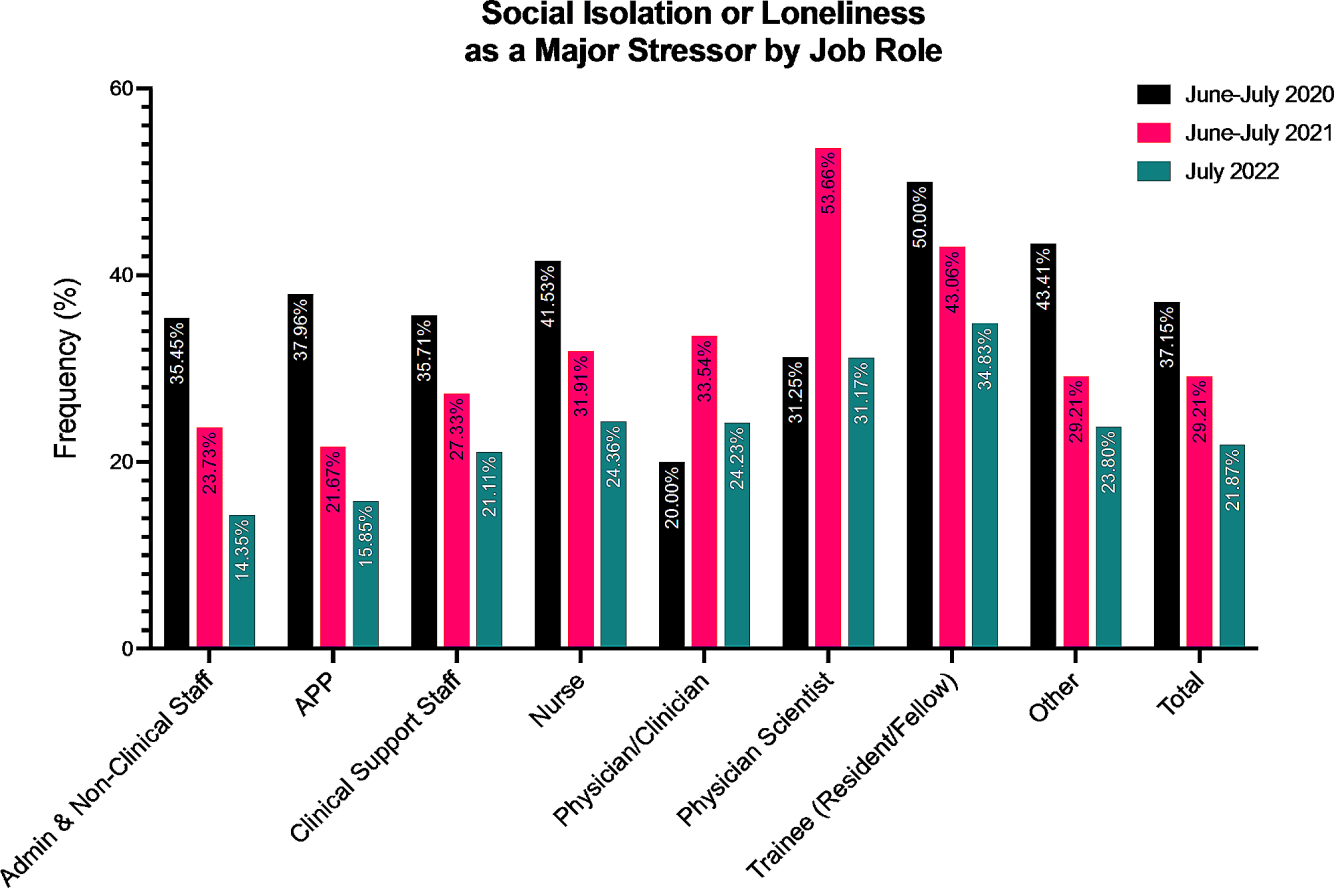
Social isolation or loneliness as a major stressor by job role

Table 2 describes social isolation or loneliness as a major stressor across job role, work location, and demographics. Job role was significantly associated with loneliness as a major stressor (*p* < 0.001). Trainees (39.2%) and Physician Scientists (38.1%) reported the highest overall percentages of social isolation or loneliness as a major stressor, followed by nurses (28.2%). The lowest percentages of loneliness were reported by clinical support staff (23.5%) and administrative or non-clinical staff (19.5%). In 2020, the highest percentages of loneliness were observed in trainees (50.0%), followed by other job roles (43.4%) and nurses (40.0%). In 2021, physician scientists (43.1%) and trainees (43.1%) reported the highest levels of loneliness as a major stressor. In 2022, trainees (43.8%) and physician scientists (31.2%) remained the most frequent reporters of loneliness across job roles. Overall, a gradual decrease in the reporting of social isolation or loneliness as a major stressor was reported over time, with the exception of the physician scientist, who reported a large increase in this stressor in 2021, though it was attenuated by 2022. A graphical representation of this data can be found in Fig. 1.

**Table 2:** Self-report of social isolation or loneliness as a major non-work stressor by demographic identifiers (*n* = 6,310)

	Social Isolation or Loneliness as a Major Stressor [Freq(%)]				*p*	
	2020	2021	2022	All		
Job Role					0.000	***
Administrative, Non-Clinical Staff	39(35.5)	84(23.7)	91(14.4)	214(19.5)		
APP	52(38.0)	26(21.7)	26(15.9)	104(24.7)		
Clinical Support Staff	20(35.7)	44(27.3)	114(21.2)	178(23.5)		
Nurse	40(40.0)	107(33.5)	174(24.2)	321(28.2)		
Physician or Clinician	26(24.8)	69(28.3)	81(23.1)	176(25.2)		
Physician Scientist	5(31.3)	22(53.7)	24(31.2)	51(38.1)		
Trainee (Resident/Fellow)	5(50.0)	31(43.1)	31(34.8)	67(39.2)		
Other	79(43.4)	177(29.2)	263(23.8)	519(27.4)		
Work Location					0.000	***
Administration/Office	70(37.6)	160(28.4)	197(18.8)	427(23.7)		
Ambulatory/Outpatient	42(36.2)	126(24.7)	114(16.8)	282(21.6)		
Hospital-Based ICU	21(35.0)	43(36.4)	75(28.9)	139(31.7)		
Hospital-Based Non-ICU	57(41.6)	93(35.1)	128(26.2)	278(31.2)		
OR/Surgical/Procedural	12(18.5)	11(14.1)	39(21.8)	62(19.3)		
Other	64(42.1)	127(33.3)	251(24.6)	442(28.4)		
Gender					0.000	***
Male	53(31.9)	146(29.7)	211(22.6)	410(25.8)		
Female	211(38.6)	406(28.7)	587(21.5)	1,204(25.7)		
Non-binary or self-describe	2(66.7)	8(88.9)	6(46.2)	16(64.0)		
Race					0.000	***
White	217(37.7)	435(29.7)	596(23.1)	1,248(27.0)		
Black or African American	25(30.5)	58(24.3)	108(16.2)	191(19.4)		
Hispanic/LatinX	6(46.2)	14(29.8)	25(29.1)	45(30.8)		
Asian/Pacific Islander	11(37.9)	30(29.4)	44(20.6)	85(24.6)		
Other/Self-Identify	7(41.2)	23(37.1)	31(24.6)	61(29.8)		
Age					0.000	***
18–24	13(59.1)	43(55.8)	87(38.5)	143(44.0)		
25–34	87(47.8)	184(35.5)	277(26.4)	548(31.3)		
35–44	75(37.1)	134(29.2)	201(22.6)	410(26.5)		
45–64	43(26.1)	106(24.3)	125(16.3)	274(20.0)		
55–65	37(35.6)	81(23.2)	94(15.4)	212(19.9)		
> 65	11(26.8)	12(15.8)	20(14.5)	43(16.9)		
Marital Status					0.000	***
Married, partnership, or cohabitating	155(29.8)	310(22.7)	429(16.5)	894(20.0)		
Non-married, partnership, or cohabitating	111(56.6)	250(45.4)	375(34.6)	736(40.2)		

Note: Freq, Frequency; ICU, intensive care unit; OR, Operating room

p*<0.05, **<0.01, ***<0.001, variable vs. social isolation or loneliness across all years

Work location was significantly associated with reporting social isolation or loneliness as a major stressor (*p* < 0.001). Overall, Hospital-based ICU (31.7%) and Hospital-based non-ICU (31.2%) were observed to have the highest reporting of social isolation or loneliness as a major stressor, followed by other work locations (28.4%). In 2020, the highest rates of loneliness were reported in Hospital-based non-ICU (41.6%), administration or office (37.6%), and ambulatory or outpatient (36.2%) settings. In 2021, Hospital-based ICU (36.4%), hospital-based non-ICU (35.1%), and other (33.3%) settings reported the highest rates of loneliness, which stayed relatively consistent in 2022, though a decline in reporting loneliness as a major stressor was to a lesser degree.

The association between gender identity and reporting social isolation as a major stressor was significant (*p* < 0.001). It was observed that those who selected non-binary or self-describe for their gender description reported the highest average rates of social isolation or loneliness compared to males and females. This was also observed across individual years. In 2020, individuals who identified as non-binary or self-describe reported the highest rates of loneliness (66.7%), followed by females (38.6%) and males (31.9%). In 2021, non-binary or self-describe individuals remained the population with the highest rates of loneliness (88.9%), with males (29.7%) and females (28.7%) reporting similar levels of the stressor. Similar trends were observed in 2022, with substantial improvement in non-binary or self-describe (46.2%), male (22.6%), and females (21.5%).

A significant association between race and social isolation or loneliness as a major stressor was detected (*p* < 0.001). Overall, Hispanic/LatinX identities reported the highest average report of loneliness, followed by other or self-identify race categories (29.8%) and white (27.0%). In 2020, Hispanic/LatinX identities reported the highest levels of loneliness (46.2%), followed by Asian or Pacific Islander (37.9%) and white (37.7%). In 2021, lower reporting of loneliness was observed, though other/self-identify identities remained elevated (37.1%), followed by Hispanic/LatinX (29.8%) and white (29.7%). In 2022, Hispanic/LatinX remained at similar levels as the previous year (29.1%), followed by other/self-identify (24.6%) and white (23.1%).

Age and social isolation or loneliness as a major stressor were significantly associated (*p* < 0.001). Overall, the youngest age grouping (18–24 year old) reported the highest rates of this stressor (44.0%), followed by 25–34 year olds (31.3%). In fact, the overall reporting of loneliness was observed to be inversely proportional to age, with reports of loneliness decreasing as age increased. Across all years, the youngest age grouping reported the highest levels of loneliness as a major stressor, followed by 25–34 year olds and subsequently 35–44 year olds.

Marital status was also examined in relation to social isolation or loneliness wherein a significant association was observed ((*p* < 0.001). Overall, individuals who reported a status of non-married, partnership, or cohabitating displayed higher levels of this stressor (40.2%), compared to those who selected married, partnership or cohabitating. This remained true across individual years, though the rates of reporting social isolation or loneliness as a major stressors was observed to decrease over time in both groups.

### Relationship between stress from social isolation or loneliness and individual well-being

To assess the association of social isolation or loneliness as a major stressor with other individual experiences, we examined reporting of the stressor in relation to resilience, well-being, distress, and stress derived from job demands. Overall, it was observed that those who cited social isolation or loneliness as a major stressor reported lower resilience scores (M = 6.46) compared to those who did not (M = 6.77, *p* < 0.001).

Well-being was scored using the Well-being Index (WBI) instrument, in which scores greater than or equal to 2 indicating distress. Similar to resilience, it was observed that those who cited social isolation or loneliness as a major stressor had higher WBI scores on average (M = 2.92) compared to those who did not (M = 1.40), see Table 3. The association between WBI and this stressor was significant (*p* < 0.001) and those who reported social isolation or loneliness had scores indicative of high distress across all of the surveyed years. In 2020, while both groups scored above the distress threshold, those who reported loneliness (M = 4.67) scored much higher than those who did not (3.60). In 2021, high WBI scores were relieved in both groups, though individuals who reported loneliness as a major stressor still scored highly (M = 2.32), while those who did not were below the distress threshold on average (M = 1.07). In 2022, distress levels remained high in lonely individuals on average, while those who did not report loneliness as a major stressor remained relatively stable (M = 1.20).

**Table 3:** Comparison of resilience, distress and job demands by stress from social isolation and loneliness

	Social Isolation or Loneliness as a Major Stressor								*p*
	2020		2021		2022		All		
	Yes	No	Yes	No	Yes	No	Yes	No	
CD-RISC Composite Score (0–8) Mean(SD)	6.38(1.2)	6.76(1.2)	6.39(1.3)	6.82(1.1)	6.55(1.3)	6.74(1.20)	6.46(1.3)	6.77(1.2)	***
WBI Mean Score (-2-9) Mean(SD)	4.67(2.1)	3.60(2.3)	2.32(2.6)	1.07(2.6)	2.76(2.6)	1.20(2.6)	2.92(2.7)	1.40(2.7)	***
Increased responsibilities or job demands as a major stressor Freq(%)	145(54.5)	186(41.3)	308(55.0)	550(40.5)	217(27.0)	769(26.8)	670(41.1)	1,505(32.2)	***

Note: Freq, Frequency; CD-RISC, Connor-Davidson Resilience Scale; WBI, Well-Being Index

p*<0.05, **<0.01, ***<0.001, variable vs. social isolation or loneliness across all years

As job demands have been associated with higher frequency of distress, we also assessed increased responsibilities and job demands as a major stressor across years relative to social isolation or loneliness and found a significant association (*p* < 0.001). Overall, those who selected loneliness or social isolation as a major stressor reported higher levels of stress derived from increased job demands (41.4%), compared to those who did not (32.2%). In 2020, over half (54.5%) of those who selected social isolation or loneliness as a major stressor reported job demands as a major stressor, while 41.3% of those who did not indicate loneliness as a stressor considered their job demands to be stressful. A similar trend was observed in 2021. Stress reporting in relation to job demands was significantly reduced in 2022, with both groups citing similar rates of stress.

## Discussion

Social interactions serve as buffers to stress, resulting in lower levels of distress among individuals; this is a phenomenon often referred to as social buffering. Without social buffering, individuals are left with the consequences of feeling isolated or lonely [20].

Overall, our results suggest that certain members of the healthcare team are more vulnerable to stress from social isolation or loneliness than others. In addition to job roles, we observed significant differences based on work location, as well as age, race, and gender. Consistent with prior research, we also identified a significant association between stress resulting from social isolation or loneliness and overall distress, decreased resilience, and increased stress derived from increased job demands [21, 22]. Rates of stress from social isolation or loneliness declined over the course of the pandemic, but remained elevated for some categories of employees. This underscores the prolonged effect of the COVID-19 pandemic on the healthcare workforce. Even in 2022, when restrictions lifted and society reopened, healthcare workers may have continued to self-isolate based on the ongoing exposure to severe COVID-19 illnesses within the healthcare setting. The contrast between continuing to care for critically ill patients within the healthcare system and witnessing society at large fully reopen may have led to increased feelings of isolation, creating the perception that healthcare workers are living in a different reality than everyone else.

### Job role & work location

We observed significant differences in social isolation or loneliness stress in both job roles and work locations. Our findings suggest that trainees are at a greater risk of experiencing stress from social isolation or loneliness. Trainees often relocate from other institutions and cities, which may result in having fewer social resources in their current place of residence. Furthermore, trainees frequently rotate to different locations within a healthcare system, which can make it challenging to establish connections with coworkers or a feel a sense of belonging in a unit or clinic. Rates of reported loneliness in trainees may in part be attributed to being less acclimatized to the healthcare setting compared to other healthcare workers who have developed work-life integration [23]. For trainees working in a laboratory setting, the closure of research labs during the pandemic may have compounded their feelings of isolation compared to those who continued to work in person.

Physician-scientists also faced a high risk of stress from social isolation or loneliness compared to other groups. The role of a physician scientist encompasses both physician and researcher responsibilities, with high expectations to excel in both roles, often without sufficient protected time for either [24]. The increased workload from both clinical and research responsibilities brought on by COVID-19 may have affected their ability to connect with others, which is further supported by our finding that stress from increased job demands was positively associated with stress from social isolation or loneliness. Additionally, because physician scientists exist in both the research and clinical realms, they may struggle to feel a sense of camaraderie with either their physician colleagues or their research counterparts, as they do not fully belong to one group or the other. This dual role may predispose them to feelings of isolation. This could be further exacerbated if the physician scientist’s research field is outside of the traditional biomedical focus, such as the social sciences [25].

The surprising finding that both hospital-based ICUs and hospital-based non-ICU settings experienced the greatest stress from social isolation or loneliness can be attributed to several possible factors. During the pandemic, many in-person services were canceled or shifted to telehealth in the ambulatory and operating room settings. Hence, one might expect that people in operating rooms or ambulatory settings would be at a higher risk of social isolation or loneliness. Contrarily, we found that those who consistently worked in teams in-person at the same location experienced the greatest stress from loneliness and social isolation. Several potential explanations may exist for this unexpected finding. First, the acuity of patients in these settings may have made healthcare workers more concerned about their exposure to the virus, prompting them to isolate more consistently outside of work. Many healthcare workers were reported to have stayed in hotels, slept in their garages, and isolated from their families early in the pandemic to avoid bringing the virus home [26, 27]. Second, it may be possible that, in the later stages of the pandemic, the continued exposure to inpatient COVID-related patient complications and death, in conjunction with society reopening, created a sense that these healthcare workers were out of alignment from their broader communities. This juxtaposition alone can create an isolating feeling.

The intensity of the work and workload in different work locations may help explain this relationship. Our results demonstrate that those indicating increased responsibilities or job demands as a major stressor were also more likely to report stress from social isolation or loneliness. When the pace or intensity of work is high, as is common in inpatient settings, people may not feel they have the opportunity to connect with their colleagues. For example, healthcare workers working long shifts with few breaks may not have felt they could offer peer support, check in with colleagues, or talk about life outside of work. Additionally, as the use of technology in work continues to increase, the lines between job and life responsibilities may be blurred, thwarting the ability to connect to others both at work and at home [28], leading to increased feelings of loneliness and isolation.

### Demographic characteristics

Our findings that the youngest employees were at the greatest risk of social isolation or loneliness align with other research highlighting a loneliness epidemic in younger generations [29]. We noted a consistent relationship between increased age and decreased stress from social isolation or loneliness. One possible explanation is that older individuals may have longer and more established relationships in their job and personal communities, providing support during challenging times. Our finding that those who were married, in partnership or cohabitating were less likely to report stress from loneliness or social isolation lends support to this notion. However, it’s important to note that other research has found that older populations are also at increased risk of social isolation or loneliness, though our data did not reflect that trend. It may be that people in the oldest age categories are not represented in our data as our respondents only included those currently working. Alternatively, as people age, they may develop more experience with coping with periods of isolation and loneliness that have occurred throughout their lifetime, making them better equipped to handle a shock to their social connections, such as the one the pandemic provided. This may lead to reduces stress during periods of isolation.

Individuals identifying their gender as non-binary or self-described reported the highest rates of loneliness. This may be due to a lack of sufficient diversity in the workplace, making it difficult for them to connect with others who share their identity. This lack of representation may predispose this group to feelings of isolation and loneliness, which were further exacerbated during the COVID-19 pandemic. This aligns with prior research indicating that individuals with marginalized gender identities may be at greater risk for a variety of negative outcomes, including violence, anxiety, depression, and psychological distress [30–32]. Similarly, healthcare workers indicating Hispanic or Latinx as their race, as well as those in smaller racial categories, (Native American or Alaskan Native, Middle Eastern, and Multiracial which were grouped into other due to a low sample), reported high rates of social isolation or loneliness. This may indicate that the organization lacks sufficient diversity to allow these individuals to find a sense of belonging and connection with others that have similar racial and ethnic experiences. Collectively, these results suggest that by building a more diverse workforce, organizations may also create a better opportunity for individuals from marginalized identities to find social support at work.

### Practical implications

Given that social connection is a crucial resource for supporting health and well-being [33] and the recent US Surgeon General recommendation of increasing social connection as a public health priority [34], organizations have an opportunity to establish mechanisms that enable people to build relationships. First, organizations must set norms of behavior with accountability for leaders and individuals who exhibit toxic or abusive behaviors, engage in gossip, or employ harmful ways of interacting. Such behaviors reduce the likelihood that people will find social support at work, impeding a sense of belonging and connection and potentially leading to maladaptive behaviors among team members. Establishing norms of behavior to guide interactions and for employee and leader selection and promotion is an important condition for fostering social connectivity. In addition to fostering a healthy culture that supports building relationships, organizations can create connection mechanisms, especially for marginalized groups. Our data corroborates that individuals from marginalized gender and racial groups may have greater difficulty with social isolation or loneliness. Forming employee resource groups may be a helpful mechanism for allowing individuals from these marginalized groups to connect more broadly across the organization [35]. Even if someone cannot find someone with a similar identity in their specific unit or department, they may still benefit from connecting institutionally for mutual support. That said, it is important to note that people have differing needs for social interaction and different responsibilities outside of work. When participation in work-related social events is seen as mandatory, it may be viewed negatively for those that have lower needs for social connection. Furthermore, if these are scheduled outside of working hours, they may exclude those with family or caregiving obligations. Keeping options voluntary and making them available during working hours may alleviate these concerns. Lastly, organizations must work to reduce any non-value-added or unnecessary work and ensure appropriate staffing, so the pace of work allows people an opportunity to foster connections with each other. This is especially important in the wake of the pandemic, with high reported rates of prolonged distress, post-traumatic stress, and other negative impacts on the healthcare workforce [36]. These relational connections become even more important for healing and recovery.

Of note is the gap between the higher rates of loneliness experienced by certain employee subgroups and the lower relative rates experienced among administrative and non-clinical workers. This suggests that those who are often in leadership roles may experience less stress from social isolation or loneliness than those functioning in clinical roles. These individuals are often those that would be responsible for leading an effort to form employee resource groups, manage performance review processes, and establish norms of behavior. This represents a critical opportunity for non-clinical leaders to have mechanisms in place to identify pockets of social isolation or loneliness for intervention and programming as they may not experience those phenomena personally to the same degree.

### Limitations

There are a few limitations to this study. The study was conducted within one organization, and therefore the results may not be generalizable to other organizations. Because the study utilized pooled cross-sectional data, we cannot determine causality. The use of self-reported data can be vulnerable to other types of bias, including social desirability bias. The variation in response rates over time, in addition to the low response rates during the early phases of the pandemic have the potential for non-response bias. Lastly, we only report perceived stress from combined social isolation or loneliness, and not the incidence of these two distinct constructs. It is likely that the incidence of social isolation and the incidence of loneliness are higher than the reported stress from the combination of these experiences.

## Conclusion

Social connection is a crucial resource for protecting and preserving the mental health and well-being of the healthcare workforce. Healthcare workers in high intensity areas, with increased job demands and with marginalized identities may be at highest risk. Younger employees, trainees, and physician-scientists are also particularly vulnerable to stress from social isolation or loneliness, representing groups that may be ideal for more targeted organizational interventions and supports.

## Data Availability

The data utilized in this study are not publicly available in order to protect the privacy of the participants, though are available by reasonable request to the corresponding author.
